# Searching for More Effective Food Baits for Tephritid Fruit Flies (Diptera: Tephritidae): Performance of Newly Developed Vial-Lures Relative to Torula Yeast Borax

**DOI:** 10.3390/insects16010053

**Published:** 2025-01-08

**Authors:** Walther Enkerlin, Emilio Arevalo, Jose Eduardo Caballero, Thomas Fezza, Esteban Garavelli, Diana Beatriz Martinez, Pedro Alexander Rodriguez, Todd Shelly, Milthon Edgardo Thomas, Antonio Villaseñor, Salvador Flores

**Affiliations:** 1 Insect Pest Control Subprogramme, Joint FAO/IAEA Centre of Nuclear Techniques in Food and Agriculture, A-1400 Vienna, Austria; 2 Colombian Agricultural Institute (ICA), Calle 17 No. 40B-76 El Poblado, Medellín CP 30868, Colombia; emilioarevalop@gmail.com; 3 Moscamed-OIRSA Program (SENASA), Tegucigalpa 11101, Honduras; jcaballero@oirsa.org (J.E.C.); ing.thomas28@gmail.com (M.E.T.); 4 USDA-APHIS-PBARC, 64 Nowelo St., Hilo, HI 96720, USA; thomas.fezza@usda.gov; 5 National Service of Agri-Food Health and Quality of Argentina (SENASA), Avenida Paseo Colón, Buenos Aires C1063ACD, Argentina; egaravelli@senasa.gob.ar; 6 Department of Diagnosis, Surveillance and Phytosanitary Campaigns (SENASA), Tegucigalpa 11101, Honduras; diana.martinezm0127@gmail.com; 7 Laboratorio Nacional de Diagnóstico Fitosanitario C.I.Tibaitatá, Instituto Colombiano Agropecuario, ICA, Km 14 via Bogotá Mosquera, Bogotá 110221, Colombia; pedro.rodriguez@ica.gov.co; 8 USDA-APHIS, 41-650 Ahiki Street, Waimanalo, HI 96795, USA; 9 Programa Moscamed SADER-SENASICA, 16 Calle 3-38 Zona 10, Guatemala City, Guatemala; moscamed.avc@gmail.com; 10 Programa Operativo de Moscas, SADER-SENASICA, Camino a los Cacaotales S/N, Metapa de Domínguez CP 30860, Chiapas, Mexico; salvador.flores.i@senasica.gob.mx

**Keywords:** invasive insects, detection trapping, torula yeast mixture, synthetic protein baits, Central and South America, Hawaii

## Abstract

Food-baited traps are an important part of early detection programs for invasive tephritid fruit fly species, as they are attractive to both sexes of all targeted species. Torula yeast borax (TYB) mixture is a standard food bait, but its longevity is limited (1–2 weeks). Synthetic food-based lures have been developed, including ammonium acetate, putrescine, and trimethylamine. However, the different formulations of these synthetic lures vary greatly in their attractiveness and longevity. Here, we present the results of field trapping in several Central and South American countries as well as Hawaii that compared captures of *Ceratitis capitata*, *Anastrepha* spp., and *Bactrocera dorsalis* in traps baited with torula yeast borax, which was replaced weekly, versus traps baited with newly developed vial-lures, which contained the same three components noted above and were not replaced over 6–10 weeks of trapping. In all countries, captures of *C*. *capitata* in vial-lure-baited traps were equal to or greater than captures in TYB-baited traps. However, the vial-lures attracted fewer *B*. *dorsalis* than TYB, and data were inconsistent for *Anastrepha* spp. The implications of these results for large-scale detection programs are discussed.

## Simple Summary

Fruit flies of the family Tephritidae include about 200 species that are serious agricultural pests, as the females deposit their eggs in many fruits and vegetables, which are then eaten by the emerged larvae. These pests, thus, cause direct losses and restrict trade to fruit-fly-free areas. To detect invasive fruit flies, countries operate a network of traps in the environment that contain substances attractive to the flies. Food baits are an important component of these networks, but a commonly used bait (an aqueous mixture of yeast) is short-lasting and requires frequent replacement. In this study, we tested the effectiveness of a newly developed food bait termed vial-lures, consisting of a gelatinous matrix impregnated with three compounds associated with decaying protein. Field tests were conducted in several countries in Central and South America and Hawaii and compared captures using the standard yeast bait (replaced weekly) versus the vial-lures (not replaced over 6- to 10-week test intervals). Results from all locations revealed that the vial-lures performed well or better than the yeast solution in capturing the Mediterranean fruit fly. However, other important fruit fly species, such as the oriental fruit fly, were captured in greater numbers with the torula yeast solution.

## 1. Introduction

Surveillance networks for invasive tephritid fruit fly pests (Diptera: Tephritidae) rely on two types of traps: those baited with male lures and those baited with food-based attractants [1]. This dual approach is considered necessary, because male lures, while more potent than food baits, are sex-specific as well as being specific to a particular genus or a few closely related genera of fruit fly species [2]. For example, the male lure trimedlure attracts only males of the genus *Ceratitis* [3]. The value of food baits arises from their attractiveness to both sexes (with captures often showing a female bias) and nearly all species of economically important Tephritidae (e.g., [3,4,5]). Additionally, males of many tephritid species do not respond to any male lure [6], and food attractants are the sole means for detecting such species. Finally, there is evidence that, early in the flight season, female captures in food-based traps precede male captures in male lure-baited traps ([7] but see [3]), suggesting that food attractants are more likely to signal the presence of incipient populations.

Food baits were initially based on the fermentation of natural sugar sources, such as cane sugar and molasses [8]. Subsequent research investigated the impact of adding yeast to aqueous sugar solutions on their attractiveness, and in the 1940s, emphasis shifted to the development of protein-based baits, including enzymatically hydrolyzed torula yeast. Hydrolysis results in the breakdown of proteins into peptides, free amino acids, or parts thereof, such as ammonia, which were found to be attractive to tephritid species [9,10]. While different proteins were used as food baits, hydrolyzed torula yeast, which was produced in combination with borax (a preservative) in pellet form (termed TYB hereafter), was found to be a superior attractant and was adopted for use worldwide [11].

Despite its high attractiveness, the TYB mixture has two drawbacks, i.e., variability in the chemical composition of the pellets [8] and, more importantly, a relatively short period of effectiveness in the field, with replacement required every 1–2 weeks ([1,12], see [13] for exceptions). Consequently, dry, synthetic food baits were developed, with the most common formulation consisting of three components (ammonium acetate, putrescine, and trimethylamine) dispensed from a single packet (or sachet) that contained all three components or three separate packets (one per component) or two components (ammonium acetate and putrescine) in a single packet or in two separate packets. The three-component bait is regarded as a more sensitive attractant for the Mediterranean fruit fly (or medfly), Ceratitis capitata (Wiedemann), and the two-component bait is considered more attractive to *Anastrepha* spp. [8]. Field trials showed that this synthetic protein bait (termed BioLure) was as effective as TYB (e.g., [14,15] but see [16,17]); although in most studies, the synthetic baits were not weathered longer than 4 weeks ([16,18,19,20,21,22,23]; for exceptions see [17,24]).

Although effective, the use of the sachet(s) in Multilure traps (Better World Manufacturing Inc., Fresno, CA, USA), a commonly used trap for food baits, is problematic, because (i) the handling time required for proper placement in the trap is high, (ii) the sachets are subject to tearing and leakage, and (iii) the sachets often detach from the trap and drop into the liquid reservoir at the bottom of the trap (Section 2.1.1), rendering them useless [25,26]. To remedy these concerns, a polymeric, cone-shaped dispenser (Scentry Biologicals Inc., Billings, MT, USA) was developed that contained all three components used in the sachet(s) and fit into a well in the top of Multilure traps (Section 2.1.1). Unfortunately, while easy to use, this dispenser has been found to have limited field effectiveness (2–4 weeks) and low attractiveness to certain tephritid species, e.g., the oriental fruit fly, *Bactrocera dorsalis* (Hendel) and *Zeugodacus cucurbitae* (Coquillett) [27,28].

The goal of the present study was to compare the effectiveness of a newly developed dispenser for the three- and two-component synthetic food baits, termed vial-lures, with TYB in trapping pest tephritid species, with emphasis on *C*. *capitata*, but with data also gathered for *Anastrepha* spp., and the oriental fruit fly, *Bactrocera dorsalis* (Hendel). The project, coordinated by the International Atomic Energy Agency (IAEA), Vienna, Austria, included studies in four countries in Central and South America as well as Hawaii, the USA, to provide a rigorous evaluation of the vial-lures in a variety of habitats and for different tephritid species. Note that the vial-lures were comprised of the same three compounds as used in BioLure (i.e., ammonium acetate, putrescine, and trimethylamine), but given the novel formulation and dispenser, particular attention was focused on the effective field longevity of the vial-lures, with all study locations comparing the performance of weathered vial-lures against that of refreshed TYB over intervals of 8–10 weeks.

## 2. Materials and Methods

### 2.1. General Methods

#### 2.1.1. Traps

In all locations, flies were captured using Multilure traps exclusively or Multilure and Jackson traps. Multilure traps are 2-piece, plastic McPhail-like traps [1], with the top portion clear and the bottom bright yellow. The bottom of the trap holds the liquid food bait or preservative and has a central, open invagination through which food-based odors exit and the attracted insects enter. A wire hanger at the top of the trap is used to suspend the trap from tree branches. Jackson traps (Scentry Biologicals, Inc., Billings, MT, USA), which are used worldwide for monitoring tephritid fruit flies, are white, triangular (or “delta”) traps made of thick paper (12.7 by 9.5 by 8.4 cm; L–W–H) [1]. A removable insert made of the same paper as the trap body and coated on 1 side with a clear adhesive is placed on the floor of the trap to retain insects. Traps are generally suspended from tree branches using a metal hanger with a straight rod positioned under the roof along the apex of the trap.

#### 2.1.2. Lures

An aqueous solution of torula yeast borax is widely used in tephritid trapping programs worldwide [11] and was used in all locations as the standard or control treatment. Torula yeast borax solution (hereafter TYB) was prepared by suspending 1 TYB pellet (5 g; Scentry Biologicals Inc., Billings, MT, USA) per 100 mL of water, with 300 mL of the TYB mixture used per Multilure trap (except Argentina, where 3 TYB pellets were used per 250 mL of water). The TYB pellets dissolved in water act both as the attractant and the killing mechanism.

**Figure 1:**
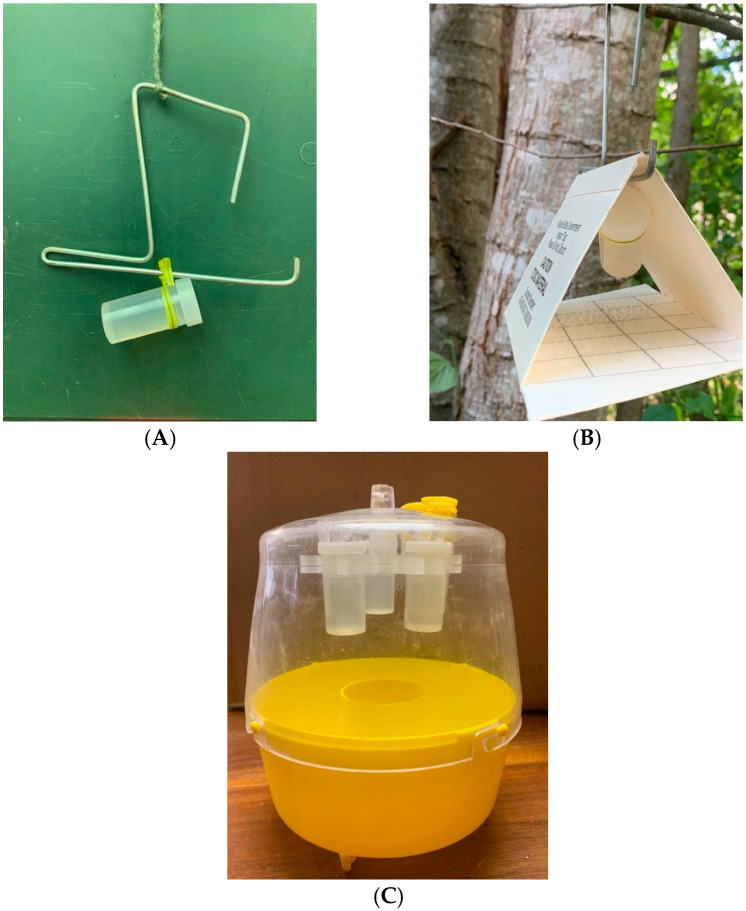
Photographs of (**A**) 3-in-1 vial-lure fastened to a metal hanger used in Jackson traps, (**B**) 3-in-1 vial-lure being placed inside Jackson trap, and (**C**) Multilure trap with 1-1-1 vial-lures held by a carousel affixed to the top inner surface of the trap.

The new synthetic food dispenser was formulated as cylindrical vial-lures (12 mm diameter; 30 mm height) that were affixed to the inner top surface of the Multilure trap or suspended in the Jackson trap from the metal hanger positioned along the apex of the trap (Figure 1). In tests involving *C*. *capitata* (and *B*. *dorsalis* in Hawaii), ammonium acetate, putrescine, and trimethylamine were either presented separately (i.e., 3 vials per trap; hereafter termed 1-1-1 vial-lures) or in combination (i.e., 1 vial per trap; hereafter termed 3-in-1 vial-lures). Based on prior studies [19,29], food baits consisting of ammonium acetate and putrescine are more attractive to *Anastrepha* spp. than the 3-component baits. Consequently, in trapping *Anastrepha*, the 2 compounds were presented together in a single vial (i.e., 1 vial per trap; hereafter termed 2-in-1 vial-lures). The alternative, i.e., presenting the 2 compounds singly in 2 different vials, was not tested. Information on the loadings of the different compounds was not available for any type of vial-lure. For all vial-lure treatments, a mixture of water and 10%-20% propylene glycol was placed at the bottom of the Multilure trap to preserve the insects and reduce evaporation [8]. As noted above, insects entering Jackson traps were retained by a sticky insert covering the floor of the trap.

#### 2.1.3. Trapping Protocol

An IAEA standardized trapping protocol was used with the exception of Hawaii [30]. Traps were placed in shaded positions in the host plant canopy and operated continuously for 8–10 consecutive weeks. In all locations, a completely randomized block design was used, with traps placed along a transect in each block with sufficient distance (15–35 m) between blocks and traps within a block. Wind direction was not considered in selecting trap sites. In all locations, traps were serviced weekly at which time (i) insects captured in Multilure traps were collected by pouring the liquid in the bottom of the trap through a sieve, (ii) TYB was replaced, (iii) the propylene glycol solution in the remaining traps was replenished (i.e., topped off at 300 mL), (iv) sticky inserts in Jackson traps were collected and new inserts were deployed, (v) trap positions were advanced one position on the transect within each block, and (vi) collected insects were transported to the laboratory to identify and count the flies.

In Hawaii, unlike the other locations, traps were not operated continuously but were deployed for 3 days at 2-week intervals. After a sampling bout, captured insects were collected, and the traps were transported to the USDA laboratory and weathered in shaded, outdoor areas under similar environmental conditions as the trapping site (Section 2.2). During weathering, the vial-lures were held in Multilure traps, but no liquid was placed in the trap reservoir. In addition, while a randomized block design was used on Oahu for trapping medflies, trapping *Bactrocera dorsalis* on Hawaii Island (commonly known as The Big Island) was conducted along a single transect at the edge of secondary forest (Section 2.2).

### 2.2. Location-Specific Methods: Study Sites, Baits Tested, Target Species, and Experimental Design

The food baits tested and the traps used at the various locations are provided in Table 1 for *C*. *capitata*, *Anastrepha* spp., and *B*. *dorsalis*. Descriptions of the study sites and additional details of the trapping protocol are given below for the different locations.

In Argentina, wild medflies were targeted, and fieldwork was conducted over 8 consecutive weeks in January–March 2020 in a mixed citrus orchard near Villa del Rosario (115 masl) in northeast Argentina. During this period, the daily minimum and maximum temperatures ranged from 15 to 21 °C and 32 to 36 °C, respectively. Traps were placed 2–3 m above ground in 5 blocks (rows) of citrus trees, with 1 trap per treatment per block (i.e., 15 total traps). Traps in adjacent blocks and adjacent positions within each block were separated by approximately 15 m.

In Mexico, testing was conducted for *C*. *capitata* over 8 consecutive weeks during May–July 2020 in the San Antonio Chicharras coffee farm located in the municipality of Tapachula, Chiapas (1134 masl). During this period, the daily minimum and maximum temperatures ranged from 16.5 to 18.3 °C and 25.2 to 27.8 °C, respectively. Captures included wild flies (both sexes) and sterile males that had been released in a Sterile Insect Technique program in this region. The study area was included in an ongoing SIT program, and 5-day-old sterile males were released weekly at a rate of 100,000 per km^2^. Trapping of *A*. *ludens* and *A*. *obliqua* was performed over 8 consecutive weeks during April–June 2020 in the mango orchard “El Rosario” located in the Canton of El Sacrificio, Chiapas (157 masl). During this period, the most extreme minimum and maximum temperatures were 24.2 °C and 32.2 °C, respectively. For both medfly and *Anastrepha* spp., a randomized block design was used, with 5 plots each containing 1 trap per treatment (i.e., 15 total traps, each 2–3 m above ground). Blocks and traps within blocks were separated by a minimum of 35 m.

In Hawaii, trapping for *C*. *capitata* was conducted biweekly over a 10-week interval during October and December 2018 in a coffee field (*Coffea arabica*; 90 masl) approximately 10 km southeast of Haleiwa, Oahu. During this period, the daily minimum and maximum temperatures ranged from 20.0 to 24.4 °C and 27.7 to 31.1 °C, respectively. Unlike the other locations, both 3-in-1 vial-lures (i.e., 1 vial-lure per trap) and 1–1-1 vial-lures (i.e., 3 vial-lures per trap) were tested in Hawaii. A randomized block design was used, with 6 blocks (rows) each containing 2 traps per treatment (i.e., 36 total traps, each 2–3 m above ground). Blocks and traps within blocks were separated by a minimum of 25 m. Unlike the other locations, trapping was not continuous but was conducted over 3-day intervals every 2 weeks over a 10-week span. Between sampling periods, the vial-lures were weathered on a covered, outside porch at the USDA Fruit Fly Laboratory, Aiea, Oahu, under conditions similar to the study site. Weathering vial-lures were held in Multilure traps (without any liquid in the trap bottom) suspended 2 m above ground from a line. Torula yeast bait was prepared just prior to use in a given trapping period.

In Hawaii, trapping for *B*. *dorsalis* was conducted biweekly over a 6-week interval during September–October 2018 along the edge of a secondary growth forest approximately 10 km south of Hilo, Hawaii Island (170 masl). Strawberry guava (*Psidium cattleyanum*), a preferred host of *B*. *dorsalis* [31], was abundant in the forest. During the study period, the daily minimum and maximum temperatures ranged from 19.3 to 22.5 °C and 25.7 to 30.1 °C, respectively. Traps baited with TYB, 3-in-1 vial-lures, or 1-1-1 vial-lures were placed alternatively along a single transect; 15 traps were deployed per treatment (45 total traps, each 1–3 m above ground), with adjacent traps separated by 20 m. After a trapping bout, vial-lures were weathered in a shaded area outside the USDA laboratory in Hilo following the procedure described above for Oahu.

In Colombia, trapping for *C*. *capitata* was conducted over an 8-week period during August–October 2020 in an orchard of *Acca sellowiana* (feijoa or pineapple guava) near the municipality of Tibasosa (2494 masl). During this period, the daily minimum and maximum temperatures ranged from 6.2 to 10.9 °C and 18.8 to 23.5 °C, respectively. Trapping for *Anastrepha* spp. was conducted over an 8-week period during October–December 2020 in a mango orchard (*Mangifera indica*) near the municipality of San Luis (620 masl). During this period, the daily minimum and maximum temperatures ranged from 19.2 to 23.1 °C and 26.6 to 31.6 °C, respectively. For all trapping, five blocks were established, with 1 trap per treatment in each (15 total traps, each 1–1.5 m above ground). Traps were placed 4 m above ground in the trees, and traps and blocks were approximately 20 m apart.

In Honduras, trapping for *A*. *obliqua* was conducted over an 8-week period during October–December 2019 in five sites, where each site represented a block, in the coastal area near the communities of Castilla, Trujillo, Santa Fe, San Antonio, and Guadalupe. The sites were within 3–10 km of one another and were climatically similar (all 10 m masl). During the study period, the daily minimum and maximum temperatures ranged from 20.5 to 25.5 °C and 26.7 to 33.9 °C, respectively. All sites contained host trees, predominantly citrus, mango (*Mangifera indica*), guava (*Psidium guajava*), and tropical almond (*Terminalia cattapa*). One trap per treatment was placed at each site (15 total traps), traps were placed 3–4 m above ground in host trees, and traps were separated by 10–15 m.

### 2.3. Statistical Analysis

Initially, the sex ratios of captured flies were compared among the different food treatments. The analysis used for a given location depended on the overall number of captures. For locations with abundant captures, sex ratios were compared on a trap-by-trap basis, restricting analysis to those traps that captured ≥8 total flies during a given sampling interval. Data were considered from all sampling intervals, and sex ratios were compared using a Kruskal–Wallis test (3-way comparisons, test statistic *H*) or a Mann–Whitney test (2-way comparisons, test statistic *U*). For locations with smaller numbers of captures, female–male proportions were compared among treatments using total counts over all sampling periods in a χ^2^ test. Independent of the particular analysis used, and for all taxa, if the variation in sex ratio was not significant, female and male data were pooled for subsequent analysis. If the variation was significant, then analysis was conducted separately for each sex.

Whether the sexes were pooled or not, subsequent analysis followed a 2-step procedure. First, a 3-way ANOVA, with main factors week, block, and bait type, examined variation in trap captures over the entire sampling interval. Capture data (x + 1) were log_10_ transformed to normalize the distribution. In most (8 of 12) of the 3-way ANOVAs conducted, the transformed data were normally distributed based on the Shapiro–Wilk test. If significant variation was detected among food bait, the Tukey test was used to identify significant differences in pairwise comparisons of group means (*p* = 0.05). Second, to more closely examine potential temporal trends in the relative performance of the different bait types, separate 1-way ANOVAs (using log_10_ x + 1) were performed for data pooled over the first and last 3 weeks of the trapping interval, respectively, followed by the Tukey test (*p* = 0.05). Reflecting the different trapping protocols (as noted above), Hawaiian data were pooled over the first and last 2 trapping events, respectively. Because the vial-lures were not replaced over the trapping period, this approach assessed whether the attractiveness of the vial-lures, relative to TYB, was similar over the trapping period or declined over time with increased weathering. Captures in vial-lure-baited traps that were statistically similar to, or even greater than, captures in torula-baited traps during the latter weeks of trapping were considered evidence of the long-lasting effectiveness of vial-lure baits.

## 3. Results

### 3.1. Ceratitis capitata

#### 3.1.1. Argentina

**Figure 2:**
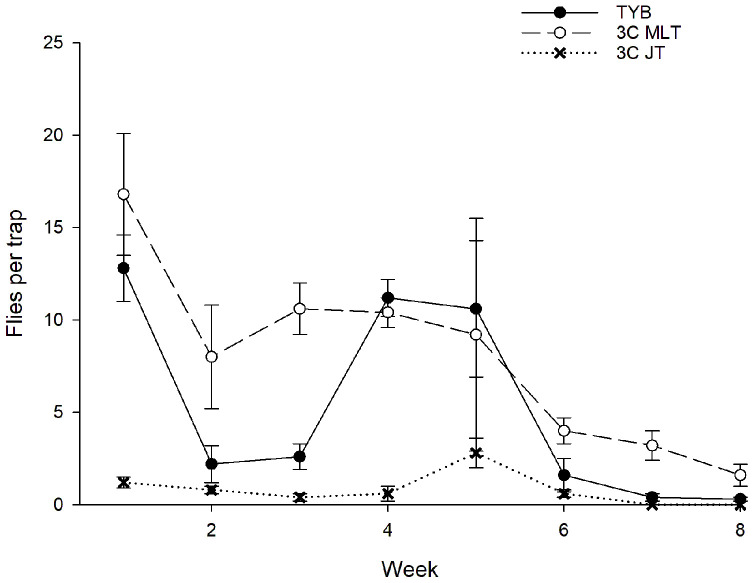
Captures of wild *C*. *capitata* (sexes combined) in traps baited with different food lures in Argentina. Symbols represent weekly averages ± 1 SE (*N* = 5 traps per food bait per week). Food bait designations: TYB, torula yeast borax; 3C MLT, 3-in-1 vial-lure in Multilure trap; 3C JT, 3-in-1 vial-lure in Jackson trap.

A total of 490 wild females and 66 wild males of medflies were captured during the study period (Figure 2). Sex ratios (% females) did not vary significantly between Multilure traps baited with TYB (average = 84%, *N* = 17) or the 3-in-1 vial-lures (average = 92%, *N* = 10; Jackson traps baited with 3-in-1 vial-lures each captured fewer than 8 flies). Captures of wild *C*. *capitata* (sexes combined) varied significantly with week (F_7, 119_ = 15.1, *p* < 0.001) and food bait (F_2, 119_ = 50.8, *p* < 0.001) but not with block (F_4, 119_ = 1.8, *p* = 0.13). The Tukey test showed that captures in Multilure traps baited with 3-in-1 vial-lures were significantly greater than those recorded for Jackson traps baited with 3-in-1 vial-lures or Multilure traps baited with TYB and that TYB-baited traps captured significantly more flies than Jackson traps baited with the vial-lures. Comparisons among food baits in the initial and final weeks, respectively, revealed significant variation for each time period (weeks 1–3: F_2, 44_ = 36.2, *p* < 0.001; weeks 6–8: F_2, 44_ = 8.8, *p* < 0.001). Importantly, post hoc analysis showed that 3-in-1 vial-lures attracted significantly more medflies than TYB in both early and later sampling intervals, i.e., the 3-in-1 vial-lures were more attractive than TYB over the entire trapping period.

#### 3.1.2. Mexico

**Figure 3:**
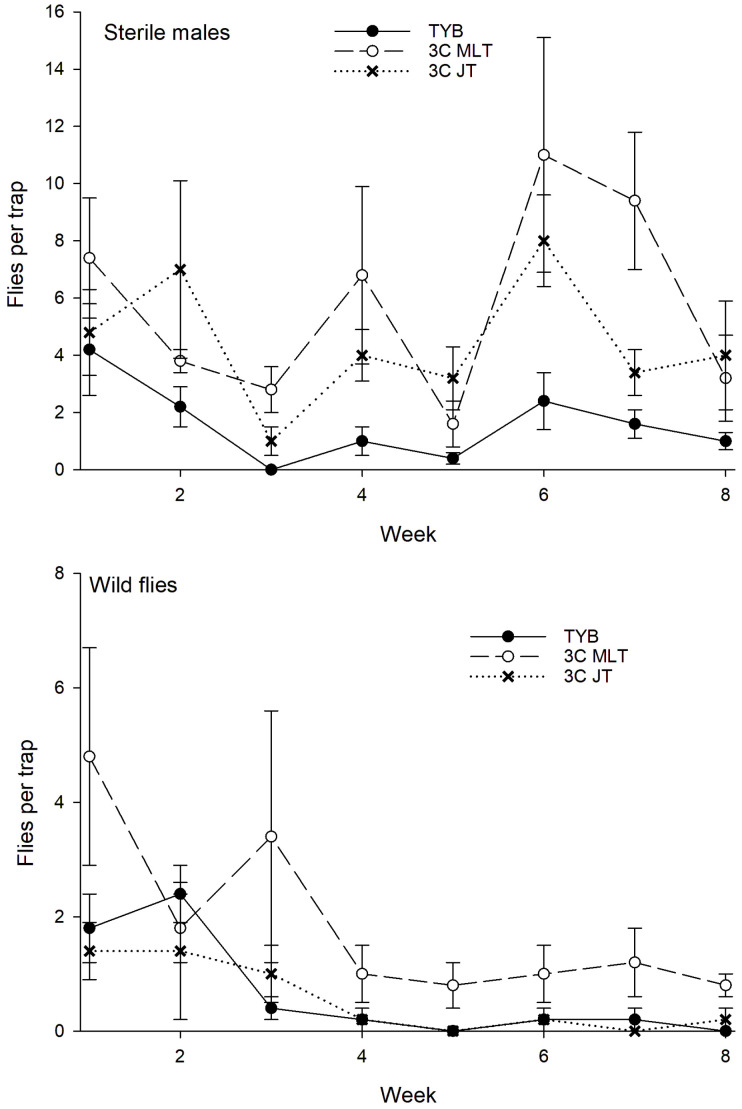
Captures of sterile males (**top**) and wild flies ((**bottom**); sexes combined) of *Ceratitis capitata* in traps baited with different food lures in Mexico. Symbols represent weekly averages ± 1 SE (*N* = 5 traps per food bait per week). Food bait designations: TYB, torula yeast borax; 3C MLT, 3-in-1 vial-lure in Multilure trap; 3C JT, 3-in-1 vial-lure in Jackson trap.

A total of 466 sterile males of *C*. *capitata* were captured during the study (Figure 3). Captures varied significantly with a week (F_7, 119_ = 7.3, *p* < 0.001) and food bait (F_2, 119_ = 19.1, *p* < 0.001) but not with a block (F_4, 119_ = 0.5, *p* = 0.71). The Tukey test showed that the 3-in-1 vial-lures in Multilure or Jackson traps captured more sterile males than TYB in Multilure traps. Captures did not differ between the Multilure and Jackson traps baited with 3-in-1 vial-lures. Comparisons among food baits in the initial and final weeks, respectively, revealed no variation for the initial weeks (weeks 1–3: F_2, 44_ = 2.6, *p* = 0.09) but significant differences for the final weeks (weeks 6–8: F_2, 44_ = 10.6, *p* < 0.001). In the later weeks, Multilure traps with the 3-in-1 vial-lures captured more sterile male medflies than TYB or 3-in-1 vial-lures in Jackson traps, while these latter two treatments did not differ significantly.

Regarding wild flies, 35 males and 87 females of *C*. *capitata* were captured (Figure 3). Sex ratios (% females) did not vary among the different food baits (torula: 17/26 = 65%; 3-in1 vial-lure in Multilure trap: 56/74 = 76%; 3-in-1 vial-lures in Jackson trap: 14/22 = 64%; χ^2^ = 1.77, df = 2, *p* = 0.41). As with the sterile males, captures of wild *C*. *capitata* varied with week (F_7, 119_ = 7.8, *p* < 0.001) and food bait (F_2, 119_ = 13.5, *p* < 0.001) but not with block (F_4, 119_ = 0.6, *p* = 0.68). The Tukey test revealed that Multilure traps baited with 3-in-1 vial-lures had higher captures of wild flies than Multilure traps baited with torula yeast or Jackson traps baited with 3-in-1 vial-lures, which did not differ from one another. Comparisons among food baits in the initial and final weeks, respectively, revealed no variation for the initial weeks (weeks 1–3: F_2, 44_ = 2.8, *p* = 0.07) but differences for the final weeks (weeks 6–8: F_2, 44_ = 10.5, *p* < 0.001). In the later weeks, Multilure traps with the 3-in-1 vial-lures captured more wild medflies than TYB or 3-in-1 vial-lures in Jackson traps, while these latter two treatments did not differ significantly.

#### 3.1.3. Hawaii

**Figure 4:**
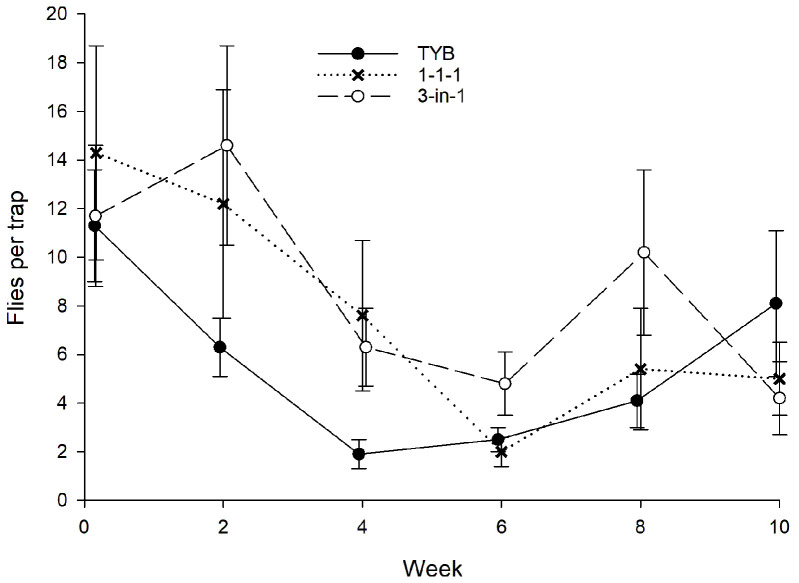
Captures of wild *Ceratitis capitata* (sexes combined) in traps baited with different food lures in Hawaii. Symbols represent averages ± 1 SE of biweekly 3-day trapping intervals (*N* = 12 traps per food bait per week). Food bait designations: TYB, torula yeast borax; 1-1-1, individual vial-lures; 3-in-1, combination vial-lures. All baits were presented in Multilure traps.

In Hawaii, a total of 1086 wild females and 505 wild males of *C*. *capitata* were captured during the study (Figure 4). Sex ratio did not vary among traps baited with the different food baits. On average, females comprised 77% of the total captures in torula yeast-baited traps (*N* = 18) compared to 67% and 68% of total captures in traps baited with 1-1-1 vial-lures (*N* = 24) and 3-in-1 vial-lures (*N* = 24), respectively (H = 5.3, *p* = 0.07). Captures of *C*. *capitata* varied with week (F_5, 215_ = 7.4, *p* < 0.001) but not with food bait (F_2, 215_ = 0.6, *p* = 0.55) or block (F_5, 215_ = 1.9, *p* = 0.10). Consistent with this result, comparisons among food baits in the initial and final weeks, respectively, revealed no significant variation for either the initial (weeks 0–2: F_2, 71_ = 0.02, *p* = 0.98) or the final (weeks 8–10: F_2, 71_ = 0.4, *p* = 0.71) weeks.

#### 3.1.4. Colombia

**Figure 5:**
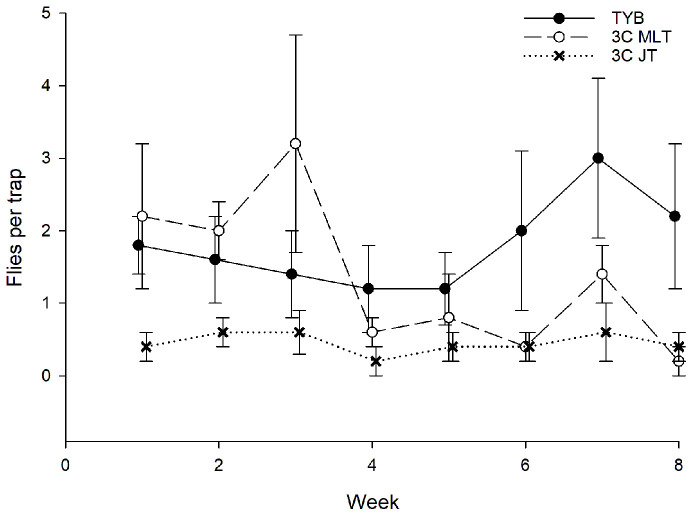
Captures of wild *Ceratitis capitata* (sexes combined) in traps baited with different food lures in Colombia. Symbols represent weekly averages ± 1 SE (*N* = 5 traps per food bait per week). Food bait designations: TYB, torula yeast borax; 3C MLT, 3-in-1 vial-lure in Multilure trap; 3C JT, 3-in-1 vial-lure in Jackson trap.

Trapping yielded a total of 38 wild males and 106 wild females of *C*. *capitata* (Figure 5). Sex ratios (% females) did not vary among the different food baits (TYB = 54/72 = 75%; 3-in-1 vial-lure in Multilure trap: 37/54 = 69%; 3-in-1 vial-lures in Jackson trap: 15/18 = 78%; χ^2^ = 1.04, df = 2, *p* = 0.60). Captures varied with food bait (F_2, 119_ = 8.7, *p* < 0.001) but not with week (F_7, 119_ = 1.3, *p* = 0.27) or block (F_4, 119_ = 1.5, *p* = 0.21). The Tukey test revealed that Multilure traps baited with TYB or 3-in-1 vial-lures had significantly higher captures than Jackson traps baited with 3-in-1 vial-lures (*p* < 0.05 in both cases). There was no difference in captures between TYB and 3-in-1 vial-lures in Multilure traps. Comparisons among food baits in the initial and final weeks, respectively, revealed variation for both the initial (weeks 1–3: F_2, 44_ = 5.4, *p* = 0.01) or the final (weeks 6–8: F_2, 44_ = 4.2, *p* = 0.02) weeks. Consistent with the above results, the Tukey test showed that 3-in-1 vial-lures and TYB attracted similar numbers of male medflies in both initial and final weeks and that Multilure traps baited with 3-in-1 vial-lures or TYB captured equal and significantly greater numbers of medflies than Jackson traps baited with 3-in-1 vial-lures in both initial and final weeks.

### 3.2. Anastrepha obliqua

#### 3.2.1. Mexico

**Figure 6:**
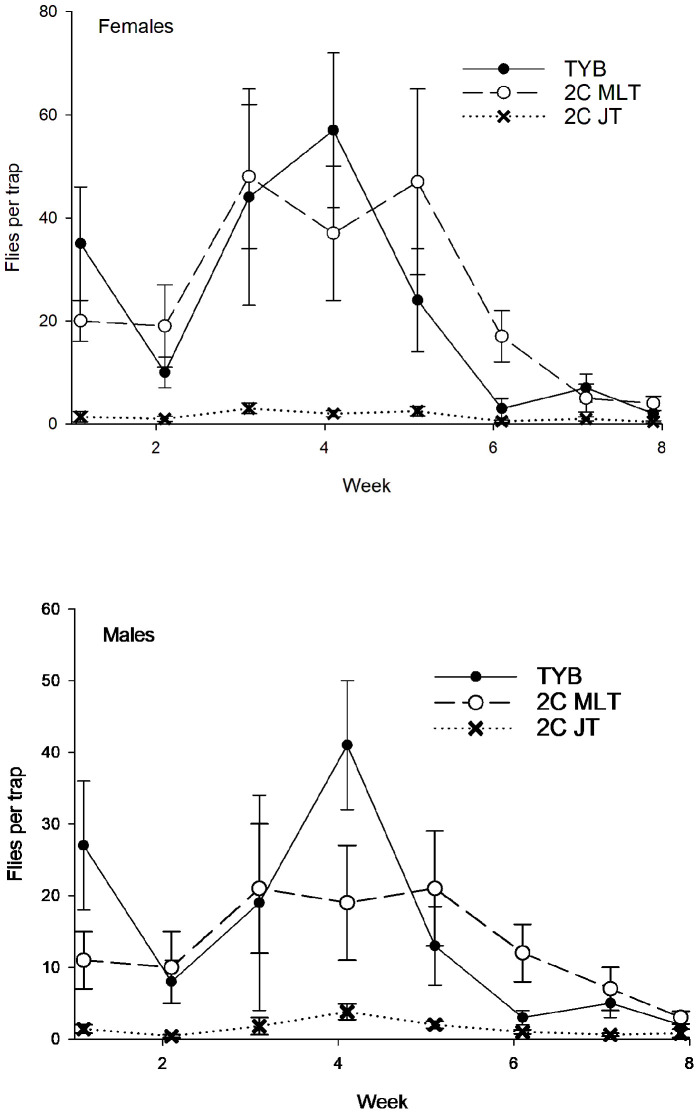
Captures of wild females and males of *Anastrepha obliqua* in traps baited with different food lures in Mexico. Symbols represent weekly averages ± 1 SE (*N* = 5 traps per food bait per week). Food bait designations: TYB, torula yeast borax; 2C MLT, 2-in-1 vial-lure in Multilure trap; 2C JT, 3-in-1 vial-lure in Jackson trap.

A total of 1955 wild females and 1146 wild males of *A*. *obliqua* were captured during the study period (Figure 6). Sex ratios (% females) varied between Multilure traps baited with TYB (average = 61%, *N* = 25) or the 2-in-1 vial-lures (average = 67%, *N* = 30; *U* = 240.5, *p* = 0.02; no Jackson traps baited with 2-in-1 vial-lures captured ≥8 flies). Captures of both *A*. *obliqua* females and males varied with week, block, and food bait (females: week, F_7, 119_ = 13.9; block, F_4, 119_ = 10.6; food bait: F_2, 119_ = 81.5; males: week, F_7, 119_ = 6.7; block, F_4, 119_ = 9.8; food bait: F_2, 119_ = 34.4; all *p* < 0.001). For both sexes, the Tukey test showed that Multilure traps baited with 2-in-1 vial-lures or TYB captured similar numbers of flies, and both of these treatments had higher catches than Jackson traps baited with 2-in-1 vial-lures. For both sexes, significant variation among treatments existed for each time period (females: early, F_2, 44_ = 23.7; late, F_2, 44_ = 11.8; males: early, F_2, 44_ = 11.9; late, F_2, 44_ = 11.5; *p* < 0.001 in all cases). For females, Multilure traps baited with 2-in-1 vial-lures or TYB, which did not differ from one another, captured more flies than Jackson traps with 2-in-1 vial-lures. This same trend was found for males in the initial weeks, but in the final weeks, Multilure traps with 2-in-1 vial-lures had higher catch than Jackson traps with 2-in-1 vial-lures or Multilure traps with TYB, and these latter two treatments did not differ from one another.

#### 3.2.2. Honduras

**Figure 7:**
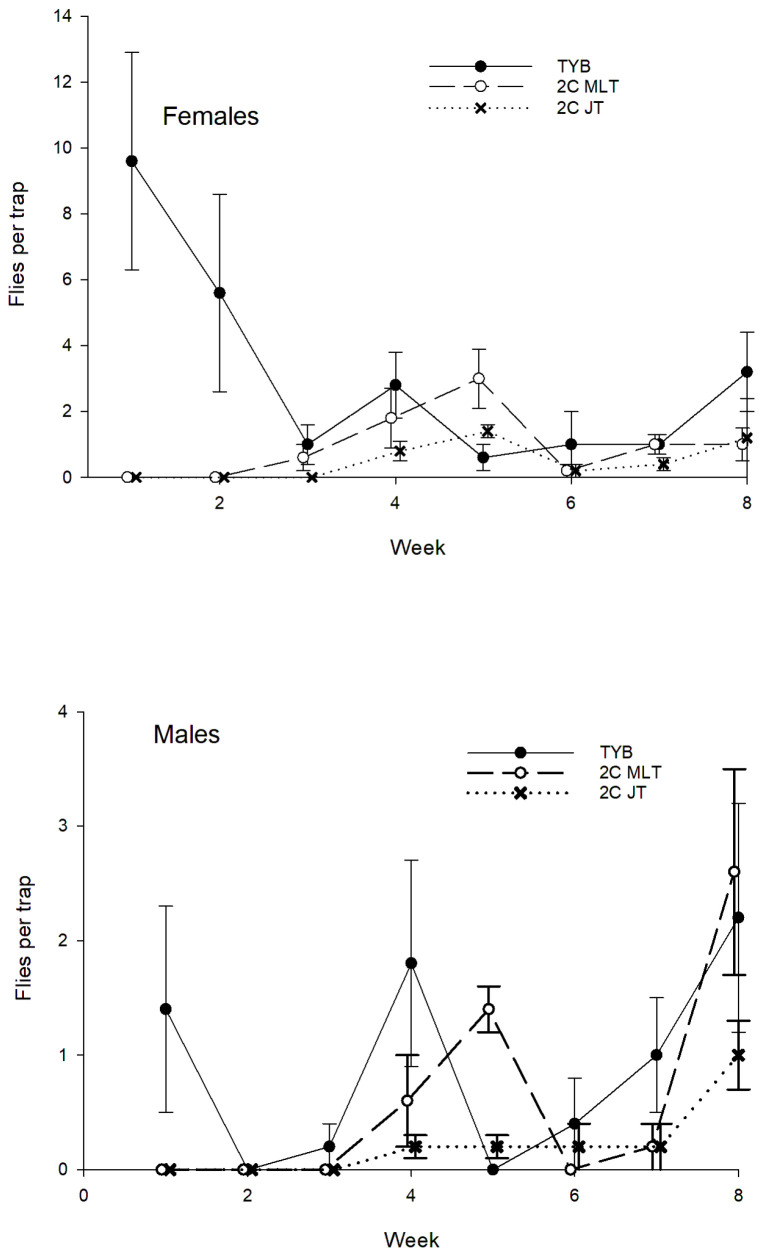
Captures of wild *Anastrepha obliqua* females (**top**) and males (**bottom**) in traps baited with different food lures in Honduras. Symbols represent weekly averages ± 1 SE (*N* = 5 traps per food bait per week). Food bait designations: TYB, torula yeast borax; 2C MLT, 2-in-1 vial-lure in Multilure trap; 2C JT, 2-in-1 vial-lure in Jackson trap.

A total of 249 wild *A*. *obliqua* were captured during the study period (Figure 7). Sex ratios (% females) varied among the different food baits (TYB = 124/160 = 77%; 2-in-1 vial-lures in Multilure traps = 38/62 = 61%; 2-in-1 vial-lures in Jackson traps = 15/27 = 55%; χ^2^ = 9.3, *p* = 0.01). Captures of *A*. *obliqua* females did not vary with week (F_7, 119_ = 1.76, *p* = 0.10) but did vary with food bait (F_2, 119_ = 8.4, *p* < 0.001) and block (F_4, 119_ = 3.9, *p* = 0.01) as two blocks accounted for 63% (111/177) of all female captures. The Tukey test showed that traps baited with TYB captured significantly more females than either vial-lure treatment or that captures with vial-lures did not differ with trap type. These same trends were found for the initial 3 weeks of trapping (F_2, 44_ = 17.2, *p* < 0.001), whereas in weeks 6–8, there was no variation among the food baits (F_2, 44_ = 0.6, *p* = 0.58), presumably reflecting the lower capture numbers during this interval. Captures of *A*. *obliqua* males varied significantly with week (F_7, 119_ = 6.4, *p* < 0.001), food bait (F_2, 119_ = 3.6, *p* = 0.03), and block (F_4, 119_ = 3.7, *p* = 0.01) as two blocks accounted for 76% (55/72) of all male captures. Traps baited with TYB captured significantly more males than Jackson traps baited with 2-in-1 vial-lures and generally more males than Multilure traps baited with 2-in-1 vial-lures, though this difference was not statistically significant. Multilure and Jackson traps baited with vial-lures captured similar numbers of *A*. *obliqua* males. In the initial 3 weeks of trapping, TYB attracted more males than 2-in-1 vial-lures in either trap type (F_2, 44_ = 3.8, *p* = 0.03), while no variation among food baits was detected in weeks 6–8 (F_2, 44_ = 0.6, *p* = 0.58).

### 3.3. Anastrepha ludens

**Figure 8:**
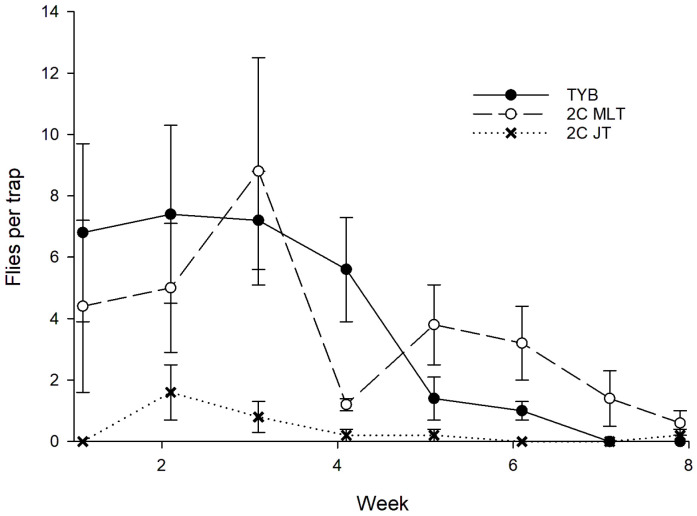
Captures of wild *Anastrepha ludens* (sexes combined) in traps baited with different food lures in Mexico. Symbols represent weekly averages ± 1 SE (*N* = 5 traps per food bait per week). Food bait designations: TYB, torula yeast borax; 2C MLT, 2-in-1 vial-lure in Multilure trap; 2C JT, 2-in-1 vial-lure in Jackson trap.

A total of 151 wild females and 153 wild males of *A*. *ludens* were captured during the study period in Mexico (Figure 8). Sex ratios (% females) did not vary among the different food baits (torula: 79/154 = 51%; 2-in1 vial-lure in Multilure trap: 66/135 = 49%; 2-in-1 vial-lures in Jackson trap: 6/15 = 40%; χ^2^ = 0.17, df = 2, *p* = 0.68). Captures of *A*. *ludens* (sexes combined) varied with week (F_7, 119_ = 8.3, *p* < 0.001), block (F_4, 119_ = 8.2, *p* < 0.001), and food bait (F_2, 119_ = 26.5, *p* < 0.001). The Tukey test showed that captures in Multilure traps baited with TYB or 2-in-1 vial-lures were greater than those recorded for Jackson traps baited with 2-in-1 vial-lures. There was no difference in catch between Multilure traps baited with 2-in-1 vial-lures or TYB. Comparisons among food baits in the initial and final weeks, respectively, revealed variation for each time period (weeks 1–3: F_2, 44_ = 11.5, *p* < 0.001; weeks 6–8: F_2, 44_ = 7.5, *p* = 0.002). Post hoc analysis showed that 2-in-1 vial-lures attracted similar numbers of *A*. *ludens* as TYB in the early sampling interval and a significantly greater number of medflies in the later interval than TYB.

### 3.4. Anastrepha spp.

**Figure 9:**
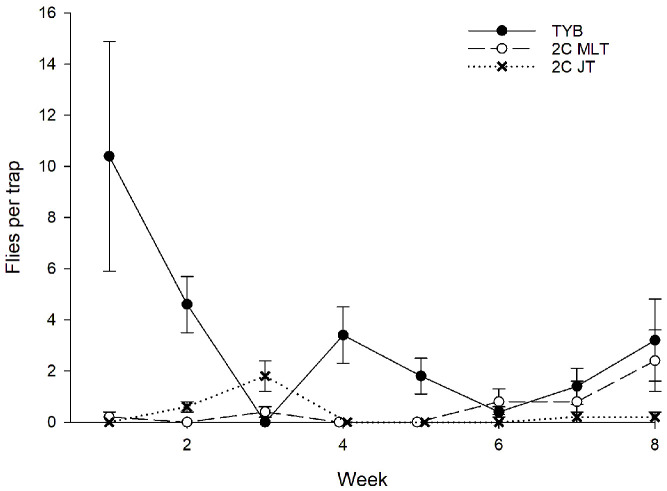
Captures of wild *Anastrepha* spp. (bottom; sexes combined) in traps baited with different food lures in Colombia. Symbols represent weekly averages ± 1 SE (*N* = 5 traps per food bait per week). Food bait designations: TYB, torula yeast borax; 2C MLT, 2-in-1 vial-lure in Multilure trap; 2C JT, 3-in-1 vial-lure in Jackson trap.

Trapping in Colombia yielded a total of 90 wild males and 73 wild females of *Anastrepha* spp. (Figure 9). Because few flies were captured in the Jackson traps, sex ratios (% females) were compared between traps baited with TYB versus traps baited with 2-in-1 vial-lures (i.e., data were pooled across Multilure and Jackson traps). This comparison showed that sex ratios did not vary among the different food baits (TYB: 58/135 = 43%; 2-in-1 vial-lure: 15/28 = 54%; χ^2^ = 1.05, df = 1, *p* = 0.34). Captures varied with food bait (F_2, 119_ = 37.9, *p* < 0.001) but not with week (F_7, 119_ = 1.9, *p* = 0.06) or block (F_4, 119_ = 1.6, *p* = 0.18). The Tukey test revealed that Multilure traps baited with TYB captured significantly more flies than 2-in-1 vial-lures in either Multilure or Jackson traps (*p* < 0.05 in both cases). Multilure and Jackson traps baited with vial-lures captured similar numbers of flies. There was also significant variation in captures in weeks 1–3 (F_2, 44_ = 9.9, *p* < 0.001) and weeks 6–8 (F_2, 44_ = 4.4, *p* = 0.01). Traps with TYB had higher captures than 2-in-1 vial-lures in Multilure traps during the initial 3 weeks, but no difference was detected between these treatments during the final 3 weeks. Captures were similar for vial-lures in Multilure and Jackson traps during both the initial and final weeks of trapping.

### 3.5. Bactrocera dorsalis

**Figure 10:**
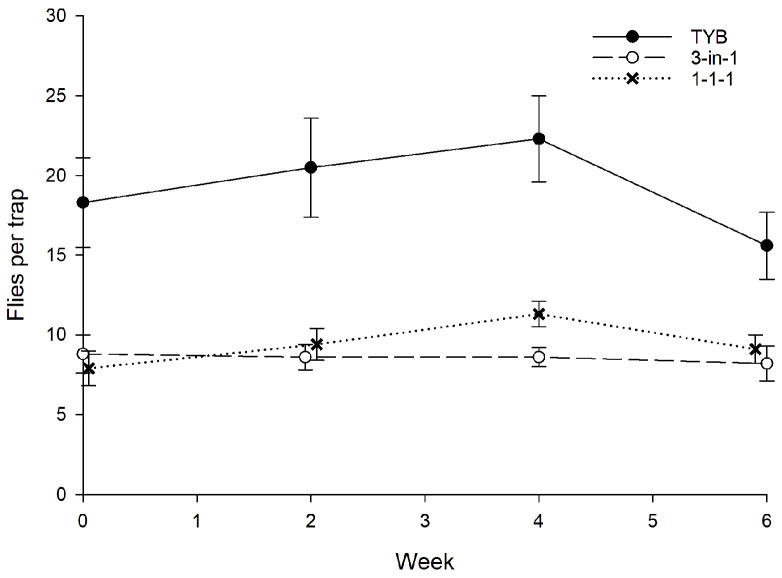
Captures of wild *Bactrocera dorsalis* (sexes combined) in traps baited with different food lures in Hawaii. Symbols represent averages ± 1 SE of biweekly 3-day trapping intervals (*N* = 15 traps per food bait per week). Food bait designations: TYB, torula yeast borax; 1-1-1, individual vial-lures; 3-in-1, combination vial-lures. All baits were presented in Multilure traps.

In Hawaii, a total of 1905 wild females and 363 wild males of *B*. *dorsalis* were captured during the study (Figure 10). Sex ratio did not vary significantly among traps baited with the different food baits. On average, females comprised 83% of the total captures in TYB-baited traps (*N* = 56) compared to 85% and 84% of total captures in traps baited with 1-1-1 vial-lures (*N* = 40) and 3-in-1 vial-lures (*N* = 33), respectively (H = 0.9, *p* = 0.65). Captures of *B*. *dorsalis* varied significantly with week (F_3, 179_ = 2.8, *p* = 0.04) and food bait (F_2, 179_ = 47.5, *p* < 0.001). Comparisons among food baits in the initial and final weeks, respectively, revealed the same pattern: There was significant variation among food baits (weeks 0–2: F_2, 89_ = 25.1, *p* < 0.001; weeks 8–10: F_2, 89_ = 22.8, *p* < 0.001), with TYB attracting significantly more flies than 3-in-1 or 1-1-1 vial-lures in both time periods. There was no significant difference between 3-in-1 and 1-1-1 vial-lures in either the early or late intervals.

## 4. Discussion

For a given location, sex ratios (% females) for *C*. *capitata* did not vary significantly among the different food bait/trap combinations. Among the three Central and South American locations, females comprised broadly similar proportions of the total samples; females constituted, on average, 65% (Mexico) to 84% (Argentina) of all captures with TYB, 69% (Colombia) to 92% (Argentina) of all captures with 3-in-1 vial-lures in Multilure traps, and 64% (Mexico) to 78% (Colombia) of all captures with 3-in-1 vial-lures in Jackson traps. Other studies of *C*. *capitata* (e.g., [20,32,33]) typically report a female bias with food-based attractants, but sex ratios may vary among different food baits. Consistent with the present study, *C*. *capitata* females comprised similar proportions of the total catch of liquid protein-baited traps and three-component BioLure traps [14,32] or three-component food cones [27]. However, Heath et al. [34], Epsky et al. [18], and Katsoyannos et al. [20] found that females comprised a greater percentage of the total catch in traps baited with synthetic food baits than TYB or NuLure (a corn-based liquid protein bait).

As with *C*. *capitata*, the sex ratio of *B*. *dorsalis* was similar between traps baited with TYB and vial-lures. In the Hawaii study, females comprised, on average, 83% of all captures with TYB, 84% of all captures with 3-in-1 vial-lures, and 85% of all captures with 1-1-1 vial-lures. Other studies of *B*. *dorsalis* generally report a female bias with food-based attractants, although female proportions are often lower and more variable than the 83%-85% range noted in the present study [3,23,27,32]. Consistent with the present study, the sex ratio of *B*. *dorsalis* did not differ between traps baited with TYB or three-component BioLure [23]. However, compared to TYB-baited traps, *B*. *dorsalis* females comprised a significantly greater proportion of the total catch in three-component food cones (approximately 80% vs. 60% for food cones and TYB, respectively) [27,28].

In contrast to the present results for *C*. *capitata* and *B*. *dorsalis*, the sex ratio was found to vary significantly among food bait/trap combinations for *A*. *obliqua* in Mexico and Honduras, respectively. However, different trends were observed in the two locations. In Mexico, females comprised a larger proportion of the total catch in traps baited with 2-in-1 vial-lures than TYB-baited traps (66% vs. 61%, respectively). In contrast, in Honduras, females comprised, on average, a much greater portion of the total catch in TYB-baited traps (77%) compared to the vial-lure-baited traps (61% and 55% for Multilure and Jackson traps, respectively). Data for *A*. *ludens* from Mexico and *Anastrepha* spp. from Colombia indicated no significant variation in sex ratio among the different food bait/trap combinations. This latter finding is consistent with other studies that showed no difference in sex ratio between TYB and two-component BioLure (*A*. *suspensa* [35]; *A*. *obliqua* [4]; *Anastrepha* spp. [21]) or food cones (*A*. *suspensa* [36]).

The performance (in terms of total captures) of the different vial-lure/trap combinations relative to the standard TYB/Multilure combinations is summarized in Table 2 for all locations and species. Several trends are noteworthy. First, regarding *C*. *capitata*, Multilure traps baited with 3-in-1 vial-lures, which were not replaced for 8–10 weeks, captured equal or greater numbers of flies as the standard TYB-baited traps, which were replaced weekly, in all four locations. The consistency of this result over different geographical regions, specific habitats, and different populations of *C*. *capitata* strongly suggests that vial-lures are effective baits for monitoring this species. This result mirrors that of an international study of BioLure sachets [18], with the important distinction that the vial-lures were weathered over 8–10 weeks compared to only 4 weeks for the BioLure formulation. Second, and again with respect to *C*. *capitata*, in all three locations where the vial-lures were placed in both Multilure and Jackson traps, (i) captures in the Multilure traps were significantly greater than those in Jackson traps and (ii) captures in vial-lure-baited Jackson traps were significantly lower than those in the standard TYB-baited traps. As dry traps, the use of Jackson traps would reduce handling time over Multilure traps, which in the present study always contained liquid (a TYB or propylene glycol solution). However, captures of *C*. *capitata* were uniformly low in vial-lure-baited Jackson traps. Interestingly, several studies [3,15,37] compared a dry food trap, namely, McPhail-type traps containing BioLure and a strip of dichlorvos as a killing agent, with standard wet food traps (McPhail-type traps with liquid protein bait) and found that captures of *C*. *capitata* in the dry traps exceeded those in the standard wet traps. The poor performance of the Jackson traps in the present study may have reflected their “openness” and associated higher escape rates compared to the closed capture volume characteristic of McPhail traps.

Results for *Anastrepha* flies revealed a third major trend (Table 2). That is, consistent with other studies, there is considerable intra- and interspecific variation in *Anastrepha* flies in the relative response to synthetic food baits versus TYB. In the present study, Multilure traps baited with vial-lures were as effective as traps baited with TYB for *A*. *ludens* and *A*. *obliqua* in Mexico. In Colombia, however, TYB-baited traps captured significantly more *Anastrepha* flies than vial-lure-baited traps, and this same result was observed for *A*. *obliqua* females in Honduras. It is not known why—in contrast to *C*. *capitata—* the results were variable for *Anastrepha*. For example, trapping was conducted in mango orchards in both Mexico and Colombia, yet 2-in-1 vial-lures (in Multilure traps) outperformed TYB in Mexico, while the reverse was observed in Colombia. Differences in host plant availability may have affected the differential response of *A*. *obliqua* females in Mexico and Honduras: Females of this species showed higher attraction to vial-lures than TYB in a guava orchard in Mexico, but the opposite result was found for females in Honduras where several different host plants were present. To our knowledge, only Martinez et al. [4] have reported comparable data for *A*. *obliqua*, and in that case, traps baited with two-component synthetic food lures captured significantly more flies than TYB-baited traps. As noted above, trapping data for other *Anastrepha* species show considerable variation. For example, catch for two-component BioLure traps exceeded that for TYB-baited traps for *A*. *ludens*, *A*. *serpentina*, and *A*. *striata*, while no difference in captures was evident between the two baits for *A*. *fraterculus* or *A*. *distincta* [4]. Regarding intraspecific variation, *A*. *ludens* has shown greater attraction to synthetic food baits in several studies [4,22,24], while greater attraction to TYB was reported in another study [17]. Similar variation characterizes *A*. *suspensa* as well [22,24,29,35,36].

Finally, although data for *Bactrocera* spp. are limited, the present result, showing greater captures in TYB-baited traps than vial-lure-baited traps, is consistent with Leblanc et al. [32] and Shelly et al. [28], who found TYB attracted more *B*. *dorsalis* than three-component BioLure or food cones, respectively.

In conclusion, this multinational research effort indicates that vial-lures that contain ammonium acetate, putrescine, and trimethylamine are deployed in Multilure traps and weathered up to 8 weeks have equal or even greater attraction to *C*. *capitata* as fresh TYB. Analysis of the initial and final weeks of trapping data for *C*. *capitata* in each location further indicates that this finding did not reflect an initial advantage of vial-lures followed by declining performance. For example, in both Argentina and Mexico, the 3-in-1 vial-lures in Multilure traps outperformed TYB during both the initial and final 3 weeks of trapping. Similarly, in Hawaii and Colombia where captures of *C*. *capitata* were similar between TYB- and 3-in-1 vial-lure-baited traps over the entire trapping period, no statistical difference was observed for the initial and final weeks, respectively. Although the data are limited, the attractiveness of vial-lures to *Anastrepha* flies appears variable. As noted, however, traps baited with synthetic food baits exhibit variable efficacy both within and among *Anastrepha* species, and more data are clearly needed to gain a rigorous assessment of vial-lures for trapping *Anastrepha* flies.

The present results indicate that—with respect to *C*. *capitata—* viallures would lengthen the replacement interval for food bait replacement and so greatly reduce both supply and labor costs associated with large-scale trapping networks. Yet, vial-lures yielded inconsistent results for *Anastrepha* flies and poor results for *B*. *dorsalis*, which highlights the need for further research to identify a food bait attractive to all species of tephritid pests.

## Figures and Tables

**Table 1:** Food baits tested in different countries for trapping *Ceratitis capitata, Anastrepha*, and *Bactrocera dorsalis.* In Mexico, the target *Anastrepha* spp. were *A*. *ludens* and *A*. *obliqua*; in Colombia, *Anastrepha* species were not distinguished; in Honduras, the target species was *A*. *obliqua*. Abbreviations: TYB, torula yeast borax mixture; 3-in-1, ammonium acetate, putrescine, and trimethylamine combined in a single vial; 1-1-1, the same 3 components held in 3 separate vials; 2-in-1, ammonium acetate and putrescine combined in a single vial; MLT, Multilure trap; JT, Jackson trap.

***Ceratitis capitata***			
**Location**	**Treatment**	**Lure**	**Trap**
Argentina	1	TYB	MLT
	2	3-in-1 vial-lure	MLT
	3	3-in-1 vial-lure	JT
Mexico	1	TYB	MLT
	2	3-in-1 vial-lure	MLT
	3	3-in-1 vial-lure	JT
Hawaii	1	TYB	MLT
	2	3-in-1 vial-lure	MLT
	3	1-1-1 vial-lure	MLT
Colombia	1	TYB	MLT
	2	3-in-1 vial-lure	MLT
	3	3-in-1 vial-lure	JT
***Anastrepha* spp.**			
**Location**	**Treatment**	**Lure**	**Trap**
Mexico	1	TYB	MLT
	2	2-in-1 vial-lure	MLT
	3	2-in-1 vial-lure	JT
Colombia	1	TYB	MLT
	2	2-in-1 vial-lure	MLT
	3	2-in-1 vial-lure	JT
Honduras	1	TYB	MLT
	2	2-in-1 vial-lure	MLT
	3	2-in-1 vial-lure	JT
***Bactrocera dorsalis***			
**Location**	**Treatment**	**Lure**	**Trap**
Hawaii	1	TYB	MLT
	2	3-in-1 vial-lure	MLT
	3	1-1-1 vial-lure	MLT

**Table 2:** Summary of performance of vial-lures relative to TYB for the different species and locations, where “+” indicates the lure/trap combination had significantly more captures than the standard TYB/MLT combination, “−” indicates the lure/trap combination had significantly fewer captures than the standard TYB/MLT combination, and “≈” indicates captures did not differ significantly from the TYB/MLT captures. Information for Mexico refers to wild *C*. *capitata* only. In Colombia, *Anastrepha* species were not distinguished; in Honduras, the target species was *A*. *obliqua*. With the exception of Honduras, capture data were pooled over the sexes as the sex ratio did not vary significantly with lure/trap combination. Abbreviations: TYB, torula yeast borax solution; 3-in-1, ammonium acetate, putrescine, and trimethylamine combined in a single vial; 1-1-1, the same 3 components held in 3 separate vials; 2-in-1, ammonium acetate and putrescine combined in a single vial; MLT, Multilure trap; JT, Jackson trap.

***Ceratitis capitata***			
**Performance Relative**			
**Location**	**Vial-Lure**	**Trap**	**to TYB/MLT**
Argentina	3-in-1	MLT	+
	3-in-1	JT	−
Mexico	3-in-1	MLT	+
	3-in-1	JT	≈
Hawaii	3-in-1	MLT	≈
	1-1-1	MLT	≈
Colombia	3-in-1	MLT	≈
	3-in-1	JT	−
***Anastrepha***			
**Performance relative**			
**Location**	**Vial-lure**	**Trap**	**to TYB/MLT**
Colombia	2-in-1	MLT	−
*Anastrepha* spp.	2-in-1	JT	−
Honduras			
*A*. *obliqua*			
females	2-in-1	MLT	−
	2-in-1	JT	−
males	2-in-1	MLT	≈
	2-in-1	JT	−
***Bactrocera dorsalis***			
**Performance relative**			
**Location**	**Vial-lure**	**Trap**	**to TYB/MLT**
Hawaii	3-in-1	MLT	−
	1-1-1	MLT	−

## Data Availability

All data supporting the conclusions of this paper are included in the paper.
